# ERM proteins support perinuclear actin rim formation

**DOI:** 10.3389/fcell.2025.1579946

**Published:** 2026-01-21

**Authors:** Yuval Hadad, Andrea Fracchia, Dagmawit Babele, Amit Ben Shushan, Gabi Gerlitz

**Affiliations:** Department of Molecular Biology, Faculty of Life Sciences and Ariel Center for Applied Cancer Research, Ariel University, Ariel, Israel

**Keywords:** calcium, cell migration, emerin, ezrin, LINC complex, moesin, nuclear envelope, radixin

## Abstract

The interaction of actin filaments with the nuclear envelope is essential for diverse cellular processes, including cell migration, nuclear positioning, and transcriptional control. The main studied mechanism that links F-actin to the nucleus is the Linker of Nucleoskeleton and Cytoskeleton (LINC) complex. Recently, the formation of a perinuclear actin rim has been identified in various cell types in response to external force or migration signals. This rim depends on the activation of the actin nucleator Inverted formin 2 (INF2) by calcium influx. However, it is unclear how the rim is coupled to the nuclear envelope. Here, we show that the nuclear membrane protein Emerin, which has an actin-binding domain, is not required for the perinuclear actin rim formation. Interestingly, we found that the Ezrin-Radixin-Moesin (ERM) proteins, known to link actin filaments to the cell membrane, are also localized to the nuclear envelope in melanoma cells. Knockdown of ERM proteins led to a reduction in the rim levels, while overexpression of ERM proteins increased the perinuclear actin rim levels. Overexpression of Ezrin also improved the rim formation in HeLa cells upon addition of a calcium ionophore. Thus, the ERM proteins appear to participate in a mechanism that links actin filaments to the nuclear envelope.

## Graphical Abstract

**Figure:**
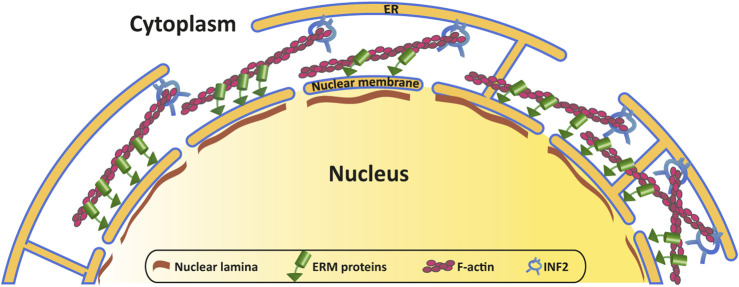

## Introduction

Interaction of the actin network with the nuclear envelope is crucial for cell migration, the correct positioning of the nucleus in polarized cells, mechanotransduction, and transcriptional control (Davidson and Cadot, 2021). One of the most recently identified nuclear envelope-associated actin structures is the perinuclear actin rim, which is also termed Calcium-mediated Actin Reset (CaAR) (Shao et al., 2015b; Wales et al., 2016; Fracchia et al., 2020; Fracchia and Gerlitz, 2022; Jessop et al., 2024; Yang et al., 2024; Labat-de-Hoz et al., 2025). The perinuclear actin rim is composed of actin filaments that engulf the nuclear envelope from its cytosolic side in both two-dimensional (2D) (Shao et al., 2015b; Wales et al., 2016; Fracchia et al., 2020; Jessop et al., 2024; Labat-de-Hoz et al., 2025) and 3D culture conditions (Fracchia et al., 2020). It was identified in various cells, including breast cancer cells, fibroblasts, epithelial cells, and melanoma cells (Shao et al., 2015b; Wales et al., 2016; Fracchia et al., 2020; Jessop et al., 2024; Yang et al., 2024; Labat-de-Hoz et al., 2025). In some cells, the perinuclear actin rim is formed transiently for 1–5 min as a reaction to an external force applied to the cell (Shao et al., 2015b; Wales et al., 2016; Yang et al., 2024). The mechanical force leads to calcium ion influx that activates the actin nucleator Inverted formin 2 (INF2) (Shao et al., 2015b; Wales et al., 2016). INF2 is a member of the formin family that supports actin polymerization, which can localize to the endoplasmic reticulum (Labat-de-Hoz and Alonso, 2020; Labat-de-Hoz et al., 2025). In epithelial cells overexpressing IFN2 or in mouse melanoma cells that are released from contact inhibition, the perinuclear actin rim is much more stable and formed for hours (Fracchia et al., 2020; Labat-de-Hoz et al., 2025).

The perinuclear actin rim was suggested to affect the migration capabilities of cells. On the one hand, enhancing perinuclear actin rim formation by adding ATP, which mediates calcium influx, accelerated the migration rate of breast cancer cells (Wales et al., 2016). On the other hand, interference with the perinuclear actin rim by expressing an active form of the actin-severing protein Gelsolin at the nuclear envelope resulted in a reduction in the migration rate of melanoma cells (Fracchia et al., 2020). In breast cancer cells, inhibition of the deacetylase Sirtuin 2 (SIRT2) resulted in the formation of a perinuclear actin rim, which was associated with a reduction in cell migration rate (Jessop et al., 2024). In search for additional factors that affect the perinuclear actin rim formation, it was found that lamin B at the nuclear side of the nuclear envelope interfered with perinuclear actin rim formation at the cytosolic side of the nuclear envelope (Fracchia et al., 2020). Perinuclear actin rim formation is independent of either the formin mDia2 (Shao et al., 2015a) or the Linker of Nucleoskeleton and Cytoskeleton (LINC) complex (Shao et al., 2015b; Fracchia et al., 2020; Jessop et al., 2024). The LINC complex is a nuclear envelope complex composed of the inner nuclear membrane SUN domain proteins that bind the outer nuclear membrane KASH domain proteins. Inside the nucleus, the SUN domain proteins interact with the nuclear lamina and chromatin, while outside of the nucleus, the KASH domain proteins can bind various cytoskeleton elements, including actin filaments (Hieda, 2019; Fracchia and Gerlitz, 2022; King, 2023; McGillivary et al., 2023).

The accumulation of the perinuclear actin rim next to the nuclear envelope led us to hypothesize that an alternative mechanism to the LINC complex anchors the perinuclear actin rim to the nuclear envelope. Here, we looked for the involvement of factors that may facilitate the connection of the perinuclear actin rim to the nuclear envelope. We found that Emerin, a nuclear envelope protein that can bind F-actin to link it to the nuclear envelope in fibroblasts and keratinocytes (Le et al., 2016; Jin et al., 2023), is not necessary for the perinuclear actin rim formation. However, Ezrin-Radixin-Moesin (ERM) proteins that are known to be involved in connecting actin filaments to the plasma membrane (Ponuwei, 2016; Pelaseyed and Bretscher, 2018; Kawaguchi and Asano, 2022; Buenaventura et al., 2023) do localize to the nuclear envelope and support the formation of the perinuclear actin rim in both mouse melanoma B16-F10 cells and human HeLa cells.

## Materials and methods

### Cell culture and transfection

Mouse melanoma B16-F10 cell line purchased from ATCC was grown as described previously (Maizels et al., 2017). Plasmids expressing GFP-fused human Ezrin, Radixin, and Moesin were a kind gift from Peter Vilmos. DNA plasmids transfection was done by the Nanojuice transfection kit (71900-3, Merck, Kenilworth, NJ, United States) and the jetOPTIMUS transfection reagent (101000025, Polyplus, Illkirch-Graffenstaden, France) following manufacturers’ instructions. Cells were incubated 24 h before further analysis. For gene silencing, cells were transfected with siRNA (IDT, Coralville, IA, United States) by the INTERFERin reagent (101000028, Polyplus). Cells were incubated for 48 h before further analysis. SiRNA used were mouse Emerin (mm.Ri.Emd.13.1), mouse Radixin (mm.Ri.Rdx.13.1), mouse Moesin (mm.Ri.Msn.13.2), mouse Ezrin (mm.Ri.Ezr.13.1) and negative control (51-01-14-04). SiRNA transfection efficiency was >90%, as verified by transfection of Cy3 Transfection Control DsiRNA (51-01-03-06). For double transfection, cells were transfected with siRNA, incubated for 24 h, transfected with DNA plasmids, and incubated for another 24 h before further analysis. 1.5 mM Ionomycin (11932, Cayman Chemical Company, Ann Arbor, MI, United States) was added for various periods.

### Immunostaining

Cells plated on Fibronectin (03-090-1-05, Biological Industries, Beit-Haemek, Israel) coated cover-glasses with or without a scratch were fixed by incubation in 2% paraformaldehyde for 10 min at room temperature or in methanol at 4 °C for 6 min. Antibodies used were rabbit anti-Emerin (sc-15378, Santa Cruz Biotechnology, Dallas, TX, United States) diluted 1:150, mouse anti-Ezrin (sc-58758, Santa Cruz Biotechnology, Dallas, TX, United States) diluted 1:200, goat anti-GFP (ab5450, Abcam, Cambridge, United Kingdom) diluted 1:400, goat anti-Lamin B (6216, Santa Cruz Biotechnology, Dallas, TX, United States) diluted 1:150, mouse anti-Moesin (CST3150, Cell Signaling Technologies, Danvers, MA, United States) diluted 1:300 and rabbit anti-Radixin (ab52495, Abcam, Cambridge, United Kingdom) diluted 1:100. Actin filaments were labeled by DyLight™ 554 Phalloidin (13054, Cell Signaling Technologies, Danvers, MA, United States) diluted 1:400. DNA was stained with Hoechst 33342 (B2261, Sigma-Aldrich, Rehovot, Israel). Images were collected using an Olympus IX81 fluorescence microscope with a coolSNAP HQ2 CCD camera (Photometrics, Tucson, AZ, United States) or a Prime BSI Express camera (Teledyne Photometrics, Tucson, AZ, United States). The ImageJ/Fiji software (National Institutes of Health, Bethesda, United States) was used to measure the mean intensities of Phalloidin or ERM proteins: either Lamin B or Hoechst signals enabled us to allocate the nucleus edge where the average signals of Phalloidin or ERM proteins was measured in each nucleus. A total average was calculated and normalized to control cells. Images were assembled with Photoshop (Adobe, San Jose, CA, United States).

### Protein lysate preparation and Western blot analysis

Cells were washed in PBS, scraped, precipitated by centrifugation at 500 *g* for 5 min at 4 °C, and sonicated in 2x SDS sample buffer (100 mM Tris pH 6.8, 10% glycerol, 2% SDS, 0.1 M DTT, and bromophenol blue) supplemented with protease inhibitor cocktail (539134, Merck, Kenilworth, NJ, United States). Samples were then heated at 95 °C for 10 min and stored at −20 °C until use. Protein extracts were separated in SDS-PAGE and analyzed by Western blot analysis using the following antibodies: rabbit anti-CTCF (3418, Cell Signaling Technology, Danvers, MA, United States) diluted 1:1,000, rabbit anti-Emerin (sc-15378, Santa Cruz Biotechnology, Dallas, TX, United States) diluted 1:5,000, mouse anti-Ezrin (sc-58758, Santa Cruz Biotechnology, Heidelberg, Germany) diluted 1:1,000, rabbit anti-histone H3 (05-928, EMD Millipore, Temecula, CA, United States), mouse anti-Moesin (CST3150, Cell Signaling Technologies, Danvers, MA, United States) diluted 1:1,000 and rabbit anti-Radixin (ab52495, Abcam, Cambridge, United Kingdom) diluted 1:1,000. Images were assembled with Photoshop (Adobe, San Jose, CA, United States).

## Results

Perinuclear actin rim was found to be connected to the nucleus in a LINC complex-independent manner in mouse fibroblasts (Shao et al., 2015b) and melanoma cells (B16-F10 cells) (Fracchia et al., 2020). The nuclear envelope protein Emerin was shown to bind actin (Lattanzi et al., 2003; Holaska et al., 2004) and to support perinuclear actin accumulation in response to mechanical strain (Le et al., 2016; Jin et al., 2023). To test the possibility that Emerin is also essential for the perinuclear actin rim in B16-F10 cells, we first examined its localization in these cells upon release from contact inhibition in the wound-healing assay. As expected, we detected Emerin mainly at the nuclear periphery and, to some extent, in the cytoplasm, in a pattern resembling the nuclear envelope and the endoplasmic reticulum (ER), respectively. This pattern of localization was reported before for Emerin (Salpingidou et al., 2007; Le et al., 2016) (Supplementary Figure 1A). The knockdown of Emerin did not affect the intensity of the perinuclear actin rims in a significant manner in B16-F10 cells (Supplementary Figures 1B-D), thus suggesting another factor links the perinuclear actin rim to the nucleus.

**FIGURE 1:**
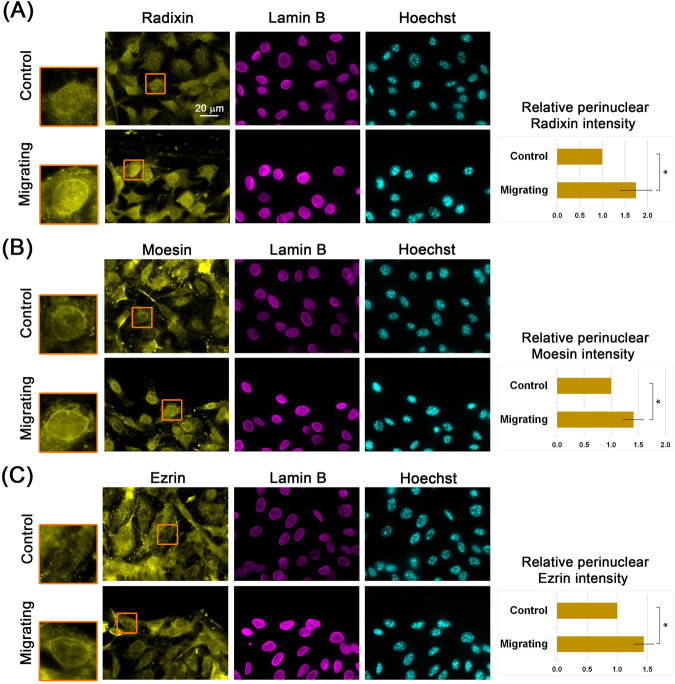
Perinuclear localization of ERM proteins. Confluent B16–F10 cells induced to migrate in the wound healing assay for 3 h, immunostained for Radixin **(A)**, Moesin **(B)**, and Ezrin **(C)**, along with Lamin B. DNA is stained with Hoechst. The edge of the scratch is in the top region of each micrograph. The nuclei in the orange rectangles are magnified. Scale bar: 20 µm. For quantification, 20–45 cells from each condition were measured in each experiment for the ERM protein signal at the nuclear periphery. The mean intensity was calculated and normalized to control cells. The average mean intensity in three independent experiments ±s.e. is presented. Statistical significance was evaluated by the Student’s t-test, *P < 0.05.

**FIGURE 2:**
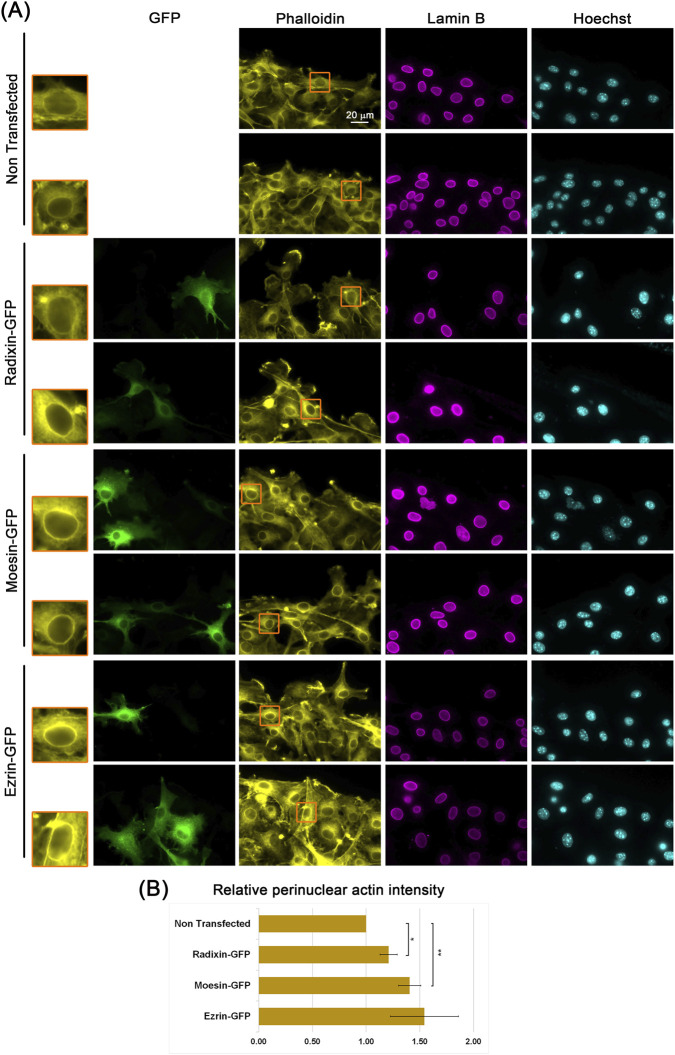
Overexpression of ERM proteins increases the intensity of the perinuclear actin rim. **(A)** Perinuclear actin rim upon overexpression of ERM proteins. Confluent B16-F10 cells over-expressing GFP-fused ERM proteins were induced to migrate in the wound healing assay for 3 h, stained for filamentous actin (Phalloidin), nuclear envelope (Lamin B), and DNA (Hoechst). The edge of the scratch is in the top region of each micrograph. The nuclei in the orange rectangles are magnified on the left side. Scale bar: 20 µm. **(B)** Quantification of the actin perinuclear rim in ERM overexpressing cells vs. control cells. For quantification, 20–30 cells from each condition were measured for the Phalloidin signal at the nuclear periphery in each experiment. The mean intensity was calculated and normalized to control cells. The average mean intensity in three independent experiments ±s.e. is presented. Statistical significance was evaluated by the Student’s t-test, *P < 0.05, **P < 0.01.

The ERM proteins are well-established factors that connect actin filaments to the plasma membrane (Ponuwei, 2016; Pelaseyed and Bretscher, 2018; Kawaguchi and Asano, 2022; Buenaventura et al., 2023). To investigate their possible involvement in perinuclear actin rim formation, we first examined their intracellular localization. Notably, we detected perinuclear accumulation of ERM proteins, which increased upon release from contact inhibition, in the wound healing assay (Figure 1). To determine if ERM proteins can influence perinuclear actin rim formation, we examined the actin rim intensity following the overexpression of ERM proteins. As shown in Figure 2, the over-expressed proteins were partially localized to the nuclear periphery. Notably, the intensity of the perinuclear actin rim increased significantly by 21% and 40% upon overexpression of GFP-fused Radixin and Moesin, respectively. This result suggests that ERM proteins can support the perinuclear actin rim.

**FIGURE 3:**
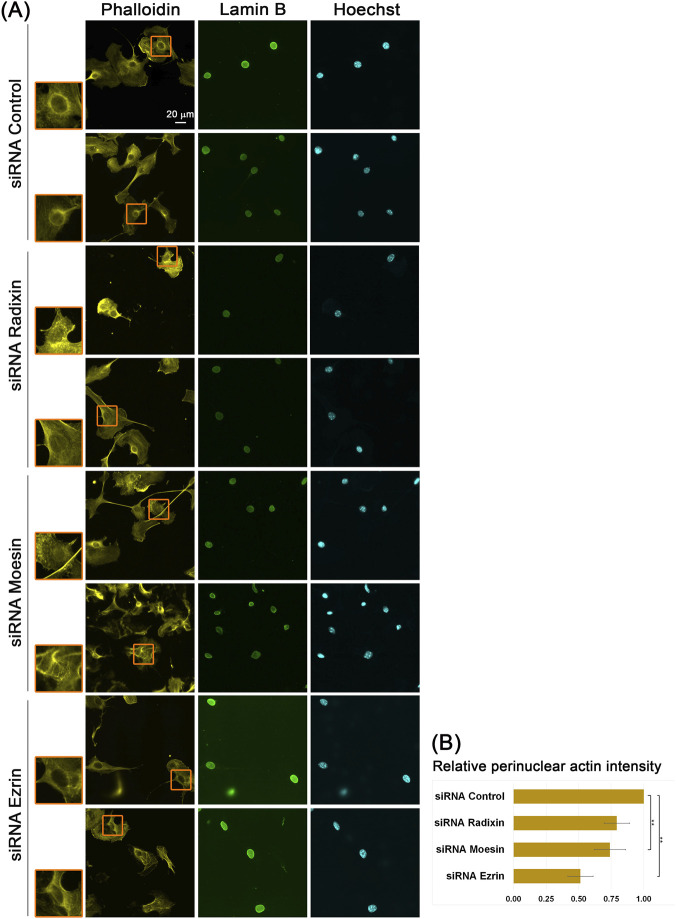
ERM proteins support the formation of a perinuclear actin rim. **(A)** Actin perinuclear rim after KD of ERM proteins. Sub-confluent B16–F10 cells transfected with either control, Radixin, Moesin, or Ezrin siRNA stained for filamentous actin (Phalloidin), nuclear envelope (Lamin B), and DNA (Hoechst). The nuclei in the orange rectangles are magnified on the left side. Scale bar: 20 µm. **(B)** Quantification of the actin perinuclear rim in siRNA ERM proteins vs. siRNA Control transfected B16–F10 cells. For quantification, in each experiment, 20–30 cells of each transfection were measured for the Phalloidin signal at the nuclear periphery. The mean intensity was calculated and normalized to control cells. The average mean intensity in three independent experiments ±s.e. is presented. Statistical significance was evaluated by the Student’s t-test, **P < 0.01.

To confirm this observation, we examined the effect of ERM proteins knockdown (Supplementary Figures 2, 3) on the formation of the perinuclear actin rim. We realized that the release of contact inhibition in the wound healing assay leads to the formation of a perinuclear actin rim, which persists as long as contact inhibition has not been restored. Therefore, at this point, we analyzed sub-confluent B16-F10 cells. SiRNA treatment reduced both the total protein amounts (Supplementary Figure 2) and their subpopulation at the nuclear envelope (Supplementary Figure 3), though not exactly to the same extent. The variances may be due to methodological differences, such as limited antigen accessibility and higher background in immunofluorescence. As seen in Figure 3, the intensity of the perinuclear actin rim was reduced by 25% and 50% following the knockdown of Moesin and Ezrin, respectively. To verify the specificity of the effect, a rescue experiment was conducted. As seen in Supplementary Figure 4, knockdown of ERM proteins led to a reduction in the perinuclear actin rim intensities, which were restored to normal levels upon overexpression of the ERM proteins in the KD cells.

**FIGURE 4:**
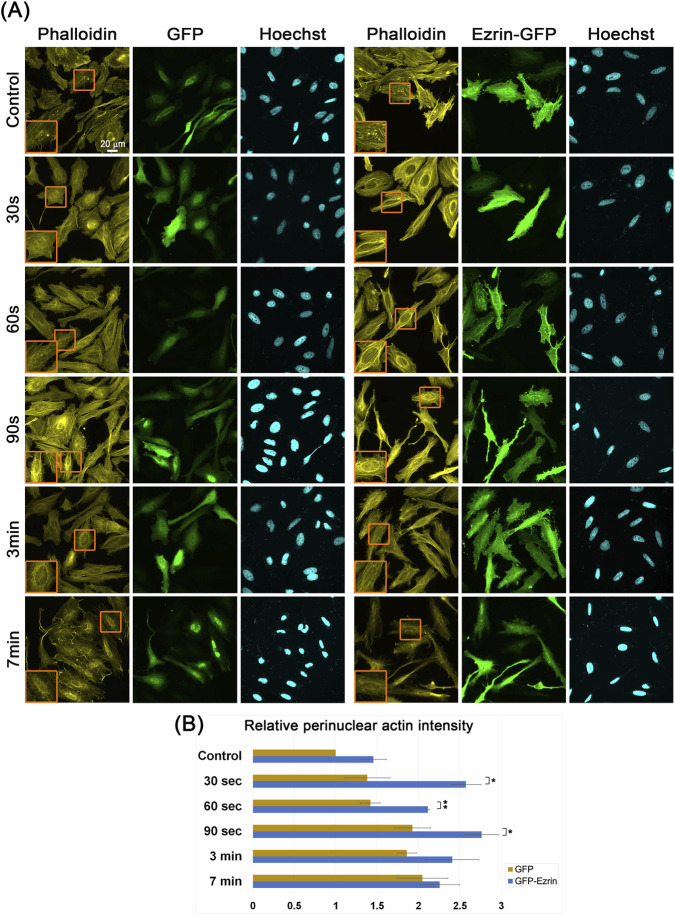
Overexpression of Ezrin increases the induction of perinuclear actin rim in HeLa cells. **(A)** Perinuclear actin rim upon ionomycin treatment in HeLa cells overexpressing Ezrin. HeLa cells overexpressing either GFP or GFP-fused Ezrin were treated for the indicated periods with ionomycin and stained for filamentous actin (Phalloidin), GFP, and DNA (Hoechst). The nuclei in the orange rectangles are magnified on the left side. Scale bar: 20 µm. **(B)** Quantification of the actin perinuclear rim in Ezrin overexpressing cells vs. GFP expressing cells. For quantification, 20–30 cells from each condition were measured for the Phalloidin signal at the nuclear periphery in each experiment. The mean intensity was calculated and normalized to control GFP-expressing cells. The average mean intensity in three independent experiments ±s.e. is presented. Statistical significance was evaluated between Ezrin-GFP and GFP-expressing cells at each time point by the Student’s t-test, *P < 0.05, **P < 0.01.

To evaluate the generality of the importance of ERM proteins in perinuclear actin rim formation, we examined the effect of Ezrin overexpression on rim formation in HeLa cells following ionomycin treatment. Previously, elevation of intracellular calcium concentration by the calcium ionophores A23187 or ionomycin was shown to induce IFN2 activity that led to perinuclear actin rim formation within seconds to minutes in various cell types, including HeLa (Shao et al., 2015b; Wales et al., 2016). Indeed, treating HeLa cells with ionomycin led to the appearance of perinuclear actin rims within seconds (Figure 4). Notably, Ezrin overexpression led to a significant increase of 43%–86% in the rim intensities at the 30-, 60-, and 90-s time points after ionomycin addition. Taken together, these results support the hypothesis that ERM proteins are part of the molecular mechanism that generates the perinuclear actin rim.

## Discussion

The perinuclear actin rim has been proposed to affect both cytoplasmic processes, such as cell migration (Wales et al., 2016; Fracchia et al., 2020; Jessop et al., 2024) and nuclear processes such as transcription (Wales et al., 2016). In search for the mechanism that links the perinuclear actin rim to the nuclear envelope, the LINC complex was evaluated. However, interference with the LINC complex function by overexpression of the KASH domain of either Nesprin 1 or Nesprin 2, which has a dominant negative effect, did not affect the formation of the actin perinuclear rim (Shao et al., 2015b; Fracchia et al., 2020). Here, we investigated the potential involvement of Emerin and ERM proteins. Emerin can localize to the outer nuclear envelope, where it was shown to bind actin filaments (Le et al., 2016), however knockdown of Emerin did not affect the perinuclear actin rim (Supplementary Figure 1).

ERM proteins can be found in the nucleus (Borkúti et al., 2024; Kovács et al., 2024), and Ezrin and Moesin were also identified in nuclear envelope fraction by a mass spectrometry analysis of purified nuclear envelopes from human leukocytes (Korfali et al., 2010). In our immunostainings, ERM proteins were partially localized to the nuclear periphery, exhibiting a similar pattern to that of lamin B immunostaining (Figures 1, 2). This localization suggests that ERM proteins can be associated not only with the plasma membrane but also with the nuclear membrane. Plasma membrane binding of ERM proteins is dependent on their FERM domain that interacts with phosphatidylinositol 4,5-bisphosphate [PI(4,5)P_2_] and on membranal proteins (Pelaseyed and Bretscher, 2018; Kawaguchi and Asano, 2022; Buenaventura et al., 2023). Notably, PI(4,5)P_2_ was found not only in the plasma membrane but also in the nuclear membrane (Smith and Wells, 1983; 1984b; 1984a; Mazzotti et al., 1995; Watt et al., 2002; Fiume et al., 2019). Thus, PI(4,5)P_2_ in the nuclear membrane could recruit ERM proteins to engulf the nucleus. An additional activator of Ezrin is S100 calcium-binding protein P (S100P), which can bind and activate Ezrin in a calcium-dependent manner (Koltzscher et al., 2003; Austermann et al., 2008). It may be relevant, since calcium influx activates the formation of the perinuclear actin rim (Shao et al., 2015b; Wales et al., 2016). Although S100P has been primarily studied in relation to the plasma membrane, it can be found in the cytoplasm at the nuclear periphery and in the nucleus (Sakaguchi et al., 2000; Maciejczyk et al., 2013).

Next, we evaluated the effect of altered levels of ERM proteins on the perinuclear actin rim. We found that knockdown of ERM proteins led to reduced levels of the perinuclear actin rim (Figure 3), which could be rescued upon overexpression of the knocked-down protein (Supplementary Figure 4). On the other hand, the overexpression of ERM proteins resulted in an increase in perinuclear actin rim levels in B16-F10 cells (Figure 2). These results suggest that all three ERM proteins contribute to the connection of actin filaments to the nuclear envelope. Due to their high similarity, they may have overlapping roles in this process. In support of the generality of this mechanism, we found that Ezrin overexpression in HeLa cells also promoted perinuclear actin rim formation in response to calcium ionophore treatment (Figure 4). The partial localization of both the endogenous and the overexpressed ERM proteins to the nuclear periphery that is also reduced by siRNA treatment suggests they may be directly involved in the perinuclear actin rim formation. Still, we cannot rule out completely the possibility that they affect the perinuclear actin rim indirectly from afar.

Combining our data with the results of others, we propose a model in which an increase in calcium ions at the nuclear periphery triggers the activation of INF2 and the polymerization of actin filaments, which are tethered by nuclear membrane-localized ERM proteins to form a rim around the nucleus. The increase in intracellular calcium ions can be due to a mechanical stimulus generated by a micromanipulation probe using atomic force microscopy (Shao et al., 2015b) or a shear stress (Wales et al., 2016). Notably, the generation of a scratch in the *in vitro* wound healing assay can generate an increase in intercellular calcium ions as well (Tran et al., 1999; Tsai et al., 2014). In some cells, the perinuclear actin rim is transient (Shao et al., 2015b; Wales et al., 2016), however, in mouse melanoma B16-F10 cells the rim is stable upon contact inhibition release as in the wound healing assay (Fracchia et al., 2020). In MDCK cells, a stable rim was also detected mainly upon mutating the WASP homology 2 (WH2) domain of INF2 (Labat-de-Hoz et al., 2025). It is still unclear which changes or factors enable long-lasting rims in some cells and not in others. Still, the perinuclear actin rim affects cellular migration and transcription (Wales et al., 2016; Fracchia et al., 2020; Jessop et al., 2024).

Here, we identify a possible new mechanism for linking actin filaments to the nuclear membrane by the ERM proteins. Our previous observation that lamin B at the inner side of the nuclear envelope can negatively affect the perinuclear actin rim formation (Fracchia et al., 2020) suggests the existence of a physical linkage between the ERM-linked actin filaments at the outer side of the nuclear envelope and the lamina at the inner side of the nuclear envelope that is still waiting to be discovered.

## Data Availability

The original contributions presented in the study are included in the article/Supplementary Material, further inquiries can be directed to the corresponding author.
