# Supplementary material for: ERM proteins support perinuclear actin rim formation
Source: Front Cell Dev Biol. 2026 Jan 21;13:1579946. doi: 10.3389/fcell.2025.1579946 (PMC12868199; doi:10.3389/fcell.2025.1579946)
Supplement: Supplementary file 1 [file DataSheet1.pdf]

Supplementary Material

Supplementary Figures

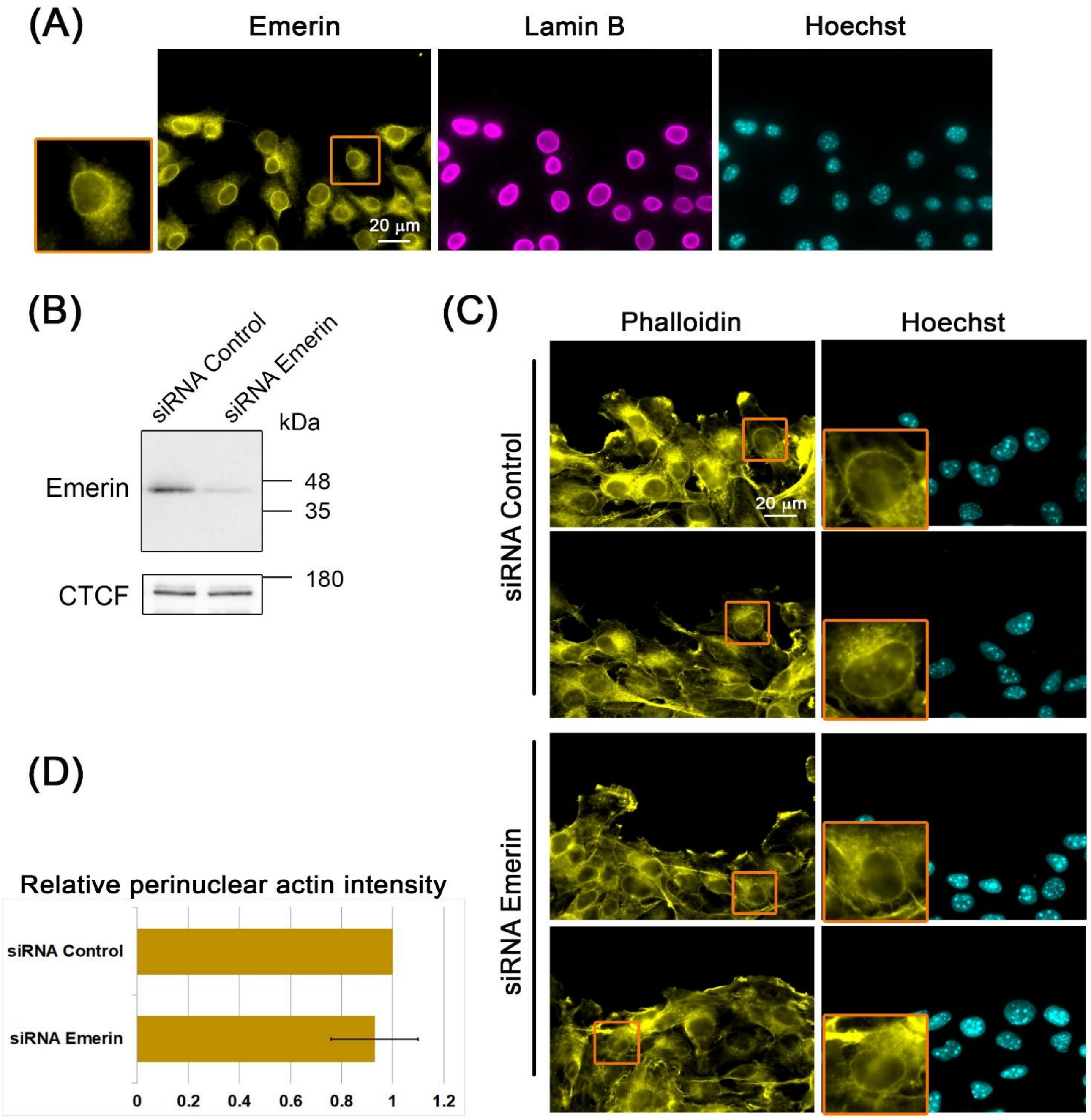

Supplementary Figure 1. **Emerin is dispensable for perinuclear actin rim formation.** (**A**) Emerin knockdown. Western blot analysis of Emerin in control and Emerin siRNA-treated B16-F10 cells. CTCF was used as a loading control. (**B**) Emerin in B16-F10 cells. Confluent B16-F10 cells induced

to migrate in the wound healing assay for 3 h immunostained for Emerin, Lamin B, and DNA (Hoechst). The edge of the scratch is in the top region of each micrograph. Scale bar: 20 µm. The nucleus in the orange rectangle is magnified on the left side. (**C**) Actin perinuclear rim in Emerin KD cells. Confluent B16–F10 cells transfected with either control or Emerin siRNA were induced to migrate in the wound healing assay for 3 h and stained for filamentous actin (Phalloidin) and DNA (Hoechst). The edge of the scratch is in the top region of each micrograph. The nuclei in the orange rectangles are magnified. Scale bar: 20 µm. (**D**) Quantification of the actin perinuclear rim in siRNA Emerin vs. siRNA Control transfected B16–F10 cells. For quantification, 40–50 cells from each condition were measured in each experiment for the Phalloidin signal at the nuclear periphery. The mean intensity was calculated and normalized to control cells. The sample difference was not statistically significant based on the Student's *t*-test.

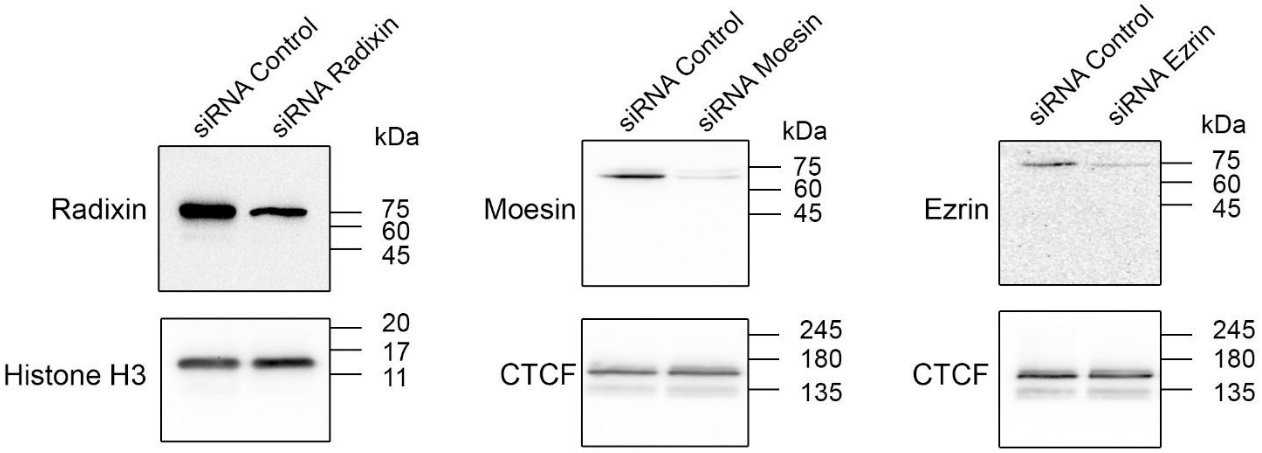

Supplementary Figure 2. **Knockdown of ERM proteins.** Western blot analysis of ERM proteins in control and Radixin/ Moesin/ Ezrin siRNA-treated B16-F10 cells. Histone H3 or CTCF was used as a loading control.

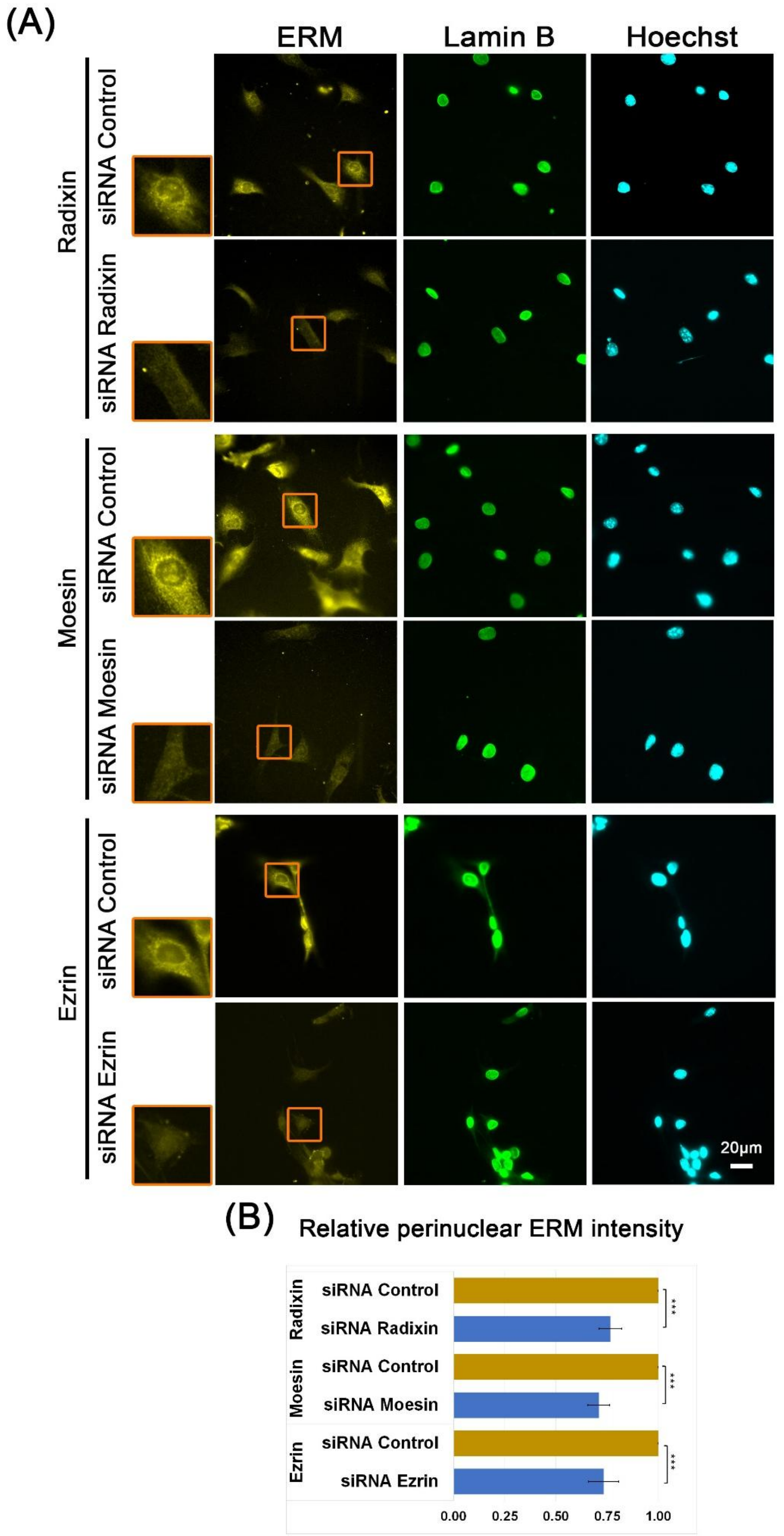

(A)
ERM
Lamin B
Hoechst
Radixin
siRNA Control
siRNA Radixin
Moesin
siRNA Control
siRNA Moesin
Ezrin
siRNA Control
siRNA Ezrin
20μm
(B)
Relative perinuclear ERM intensity
Radixin
siRNA Control
siRNA Radixin
Moesin
siRNA Control
siRNA Moesin
Ezrin
siRNA Control
siRNA Ezrin
***
***
***
0.00
0.25
0.50
0.75
1.00

◄Supplementary Figure 3. **Knockdown of ERM proteins at the nuclear periphery.** (**A**) ERM proteins immunostaining after their knockdown. ERM proteins stained in methanol-fixed sub-confluent B16–F10 cells transfected with either control or their own siRNA. Nuclear envelope (Lamin B) and DNA (Hoechst) were stained as well. Scale bar: 20 µm. (**B**) Quantification of the ERM proteins at the nuclear periphery upon their KD vs. control siRNA transfected B16–F10 cells. For quantification, in each experiment, 27-56 cells of each transfection were measured for the ERM proteins signal at the nuclear periphery. The mean intensity was calculated and normalized to control cells. The average mean intensity in three independent experiments ± s.e. is presented. Statistical significance was evaluated by the Student's *t*-test, $^{**}P<0.01$, $^{***}P<0.001$.

Supplementary Figure 4. **Overexpression of ERM proteins restores the perinuclear actin rim in ERM KD cells.** (**A**) Actin perinuclear rim in ERM proteins KD cells that overexpress either GFP or ERM proteins. Sub-confluent B16–F10 cells transfected with either control, Radixin, Moesin, or Ezrin siRNA, along with either GFP or GFP-fused ERM proteins, were stained for GFP, filamentous actin (Phalloidin), nuclear envelope (Lamin B), and DNA (Hoechst). The nuclei in the orange rectangles are magnified on the left side. Scale bar: 20 µm. (**B**) Quantification of the actin perinuclear rim in siRNA ERM proteins vs. siRNA Control transfected B16–F10 cells. For quantification, in each experiment, 20-30 cells of each transfection were measured for the Phalloidin signal at the nuclear periphery. The mean intensity was calculated and normalized to that of cells transfected with control siRNA and a GFP-expressing plasmid. The average mean intensity in three independent experiments ± s.e. is presented. Statistical significance was evaluated by the Student's *t*-test, $^{*}P<0.05$. ►

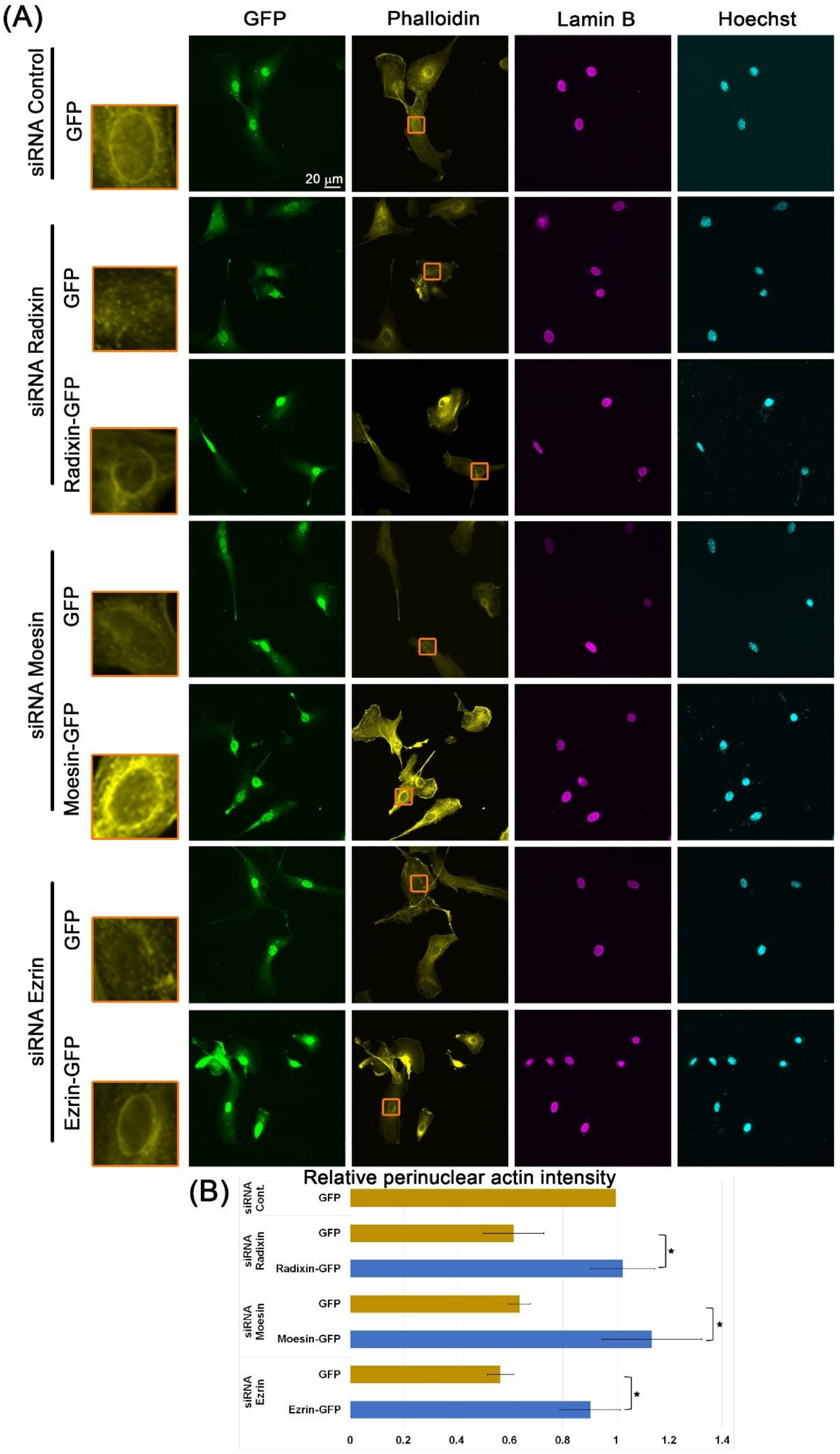
(A)
GFP
Phalloidin
Lamin B
Hoechst
siRNA Control
GFP
20 μm
siRNA Radixin
GFP
Radixin-GFP
siRNA Moesin
GFP
Moesin-GFP
siRNA Ezrin
GFP
Ezrin-GFP
(B)
Relative perinuclear actin intensity
siRNA Cont.
GFP
siRNA Radixin
GFP
Radixin-GFP
siRNA Moesin
GFP
Moesin-GFP
siRNA Ezrin
GFP
Ezrin-GFP
*
0
0.2
0.4
0.6
0.8
1
1.2
1.4
